# Neuro-hormonal Regulation Is a Better Indicator of Human Cognitive Abilities Than Brain Anatomy: The Need for a New Paradigm

**DOI:** 10.3389/fnana.2019.00101

**Published:** 2020-01-09

**Authors:** Arthur Saniotis, James P. Grantham, Jaliya Kumaratilake, Maciej Henneberg

**Affiliations:** ^1^Department of Medical Laboratory Science, Knowledge University, Erbil, Iraq; ^2^Biological Anthropology and Comparative Anatomy Research Unit (BACARU), Adelaide Medical School, University of Adelaide, Adelaide, SA, Australia; ^3^Institute of Evolutionary Medicine, Faculty of Medicine, University of Zurich, Zurich, Switzerland

**Keywords:** intelligence quotient (IQ), brain size evolution, dopamine, serotoinin, neurotrophin, enteric gut microbiome

## Abstract

Human intelligence has been theorized since the ancient Greeks. Plato and Aristotle incorporated theories of human intelligence into their metaphysical and cosmological theories which informed the social and medical sciences for centuries. With the advent of the 20th century, human intelligence became increasingly standardized based on Intelligence Quotients (IQ). Moreover, multiple theories of human intelligence were posited on morphological features of the human brain, focusing on cranial volume and size of the pre-frontal cortex which was suggestive of superior human cognitive abilities. This article argues that fixation with anatomical features of the brain was tended to ignore the importance of neuro-hormonal regulation which is a more appropriate indicator of human cognitive abilities. The article challenges the correlation between brain size and human cognitive abilities while offering an alternate theory of human cognitive abilities which emphasizes the roles of neurotransmitters, neurotrophins, and enteric gut microbiome (EGM) regulation.

## Bigger Is Not Necessarily Better: Historical Roots

The historic understanding that intelligent individuals are “big-brained” has permeated throughout modern science and prompted subsequent research to support this view. The noted neuroanatomist Paul Broca (1924–1880) suggested that bigger brain size correlated with higher intelligence and that Europeans had larger brains than Africans (Broca, 1861). Such ideas influenced scientific and political establishments during the period (Morton, 1849; Darwin, 1871).

Moreover, Galton (1888) demonstrated that university students with larger cranial measurements had an overall better academic performance. Subsequent investigations in the 20th century, in which more sophisticated methods were used to measure brain size and evaluate intelligence seemed to support the findings of Galton (Tan et al., 1999). However, a meta-analysis conducted in 1996 found that correlations between the various measures of brain size and intelligence scores ranged between 0.08 and 0.35, with an unweighted mean of 0.21 (Rushton and Ankney, 1996). Importantly, this unweighted correlation coefficient of 0.21 indicates that less than 5% of all variance in intelligence can be attributed to brain size. These findings have led many researchers to question whether there is a significant relationship between cranial volume and intelligence (Gould, 1996). Moreover, Intelligence Quotients (IQ) comprises knowledge and skills acquired through informal and formal education. Education varies depending on the socioeconomic circumstances of individuals and communities. Stature varies with socioeconomic conditions, and there is a correlation between stature and brain size. Thus, brain size/IQ covariance may be an artifact of living conditions and education (Olivier et al., 1970; Henneberg et al., 1985). Average brain sizes of females are approximately 150 ml (one standard deviation) smaller than those of males, yet there is no concomitant female/male difference in mental aptitude test scores (Henneberg et al., 1985).

These views are substantiated by numerous studies conducted that fail to show a statistically significant relationship between head size and scores on intelligence testing (Henneberg et al., 1985; Saniotis et al., 2014). The phenomenon of “publication bias” has likely prevented more publications of the negative finding of no relationship between brain size and intelligence, as authors who find no correlation tend to think that they have nothing to report. Reservations surrounding the link between brain size and intelligence are further supported by a number of contentions regarding the possibility of spurious correlations. These include body height, which is known to correlate with both cranial capacity and intelligence (Henneberg, 1998).

## Hypothesis

Although a general positive correlation between brain sizes/encephalizations and behavioral complexity exists when comparisons are made across members of various animal orders, such regularities do not seem to work in both species and individuals (Lefebvre, 2013). These inconsistencies further illustrate the limitations of an anatomical explanation in describing sophisticated phenomena, such as human cognitive abilities. The hypothesis on the positive link between intelligence and brain size in hominins has been already falsified by numerous findings. Therefore, a new hypothesis is needed. There needs to be increased attention paid to neurohormonal regulation in influencing human cognitive abilities. We offer an alternate theory of human cognitive abilities which emphasizes the roles of neurotransmitters/neurotrophins and enteric gut microbiome (EGM) regulation.

## Brain Evolution and Conceptual Challenges

Human brain evolution has for decades been informed by an essentialist approach in describing biological variation. A study of the literature reveals the maintenance of essentialist ideas in understanding brain evolution, with a principal idea of brain volume being the underlying signifier of intelligence being incorrect (Saniotis and Henneberg, 2013a).

Much of the fascination into human brain size has tended to focus on the human frontal lobe. A probable reason for this persistence with frontal lobe volume has been in order to verify the superiority of human cognitive abilities over non-human animals. Since the frontal lobe (more specifically the prefrontal neocortex) regulates executive functions and higher-order cognition it is reasonable that scientists focussed on this area. Although there has been considerable frontal lobe expansion in hominins from the early Paleolithic period, this issue has not been resolved and remains unclear (Barton and Venditti, 2013).

Consequently, two schools of thought have arisen relating to whether human prefrontal cortex (PFC) is predictably larger for a primate brain (Blinkov and Glezer, 1968; Passingham, 1973; McBride et al., 1999; Sherwood et al., 2005; Navarrete et al., 2011), or is not predictably larger for a primate brain (Brodmann, 1913; Semendeferi et al., 2002; Smaers et al., 2011; Barton and Venditti, 2013; Hoffmann, 2013). Some theorists cite lack of consistency in measuring differences in relation to white and gray matter volume in the PFC (Smaers et al., 2011), total frontal cortex size (Semendeferi et al., 2002), or inappropriate measuring methods for this lack of consensus (Elston and Garey, 2009; Barton and Venditti, 2013). For example, dimensions of various cerebral structures have altered at dissimilar levels during the evolution of body size and brain (Barton and Harvey, 2000; Barton and Venditti, 2013). Furthermore, hominin cranial material tends to be fragmentary and open to speculations. A methodological problem lies in the making of endocasts of hominin braincases. Henneberg (1998) claims that while braincase volume can be larger in hominin specimens than the tangible brain, it is the endocast that is used as an indicator of the actual brain size.

Another issue relates to a lack of vigorous research into the cytoarchitecture of the brain. An understanding into the cytoarchitecture of the PFC such as the number of neurons and cortical networks in the granular layer of the PFC (gPFC; Barton and Venditti, 2013), has implications on how intelligence is measured (Elston and Garey, 2009).

Unfortunately, the laborious nature of comparing the PFC across species, as well as, lack of scientific funding detract from a further scientific investigation in this important area of brain evolution (Elston and Garey, 2009; Passingham and Smaers, 2014).

Furthermore, in keeping with the hypothesis of brain size contributing to improved intelligence, males, with their firmly established average larger cranial capacity, should demonstrate greater intellectual aptitude. However, the sexual dimorphism of mental ability, although proposed, remains inconclusive. Perhaps the biggest detraction from considering brain volume predictive of intelligence is the weakness of the correlation. Even when accepting the findings of the most ardent supporters of brain size informing intelligence, only a modest influence can be claimed. This leads us to the conclusion that variation in mental aptitude must be primarily a result of the variation in brain physiology, especially biochemical variation in substances facilitating neuronal communication (Henneberg et al., 1985). This is further demonstrated in a recent study that found that individuals possessing higher mental performance have less neuronal arborization and density of dendrites. The study suggests that higher intelligence is predicated on less but better organized and efficient neurons.

## The Decrease in Human Brain Volume

With the broader scientific acceptance that larger brains convey greater mental capacity, numerous studies have concentrated on showing a postulated link between an evolutionary enlargement of the brain and increased behavioral complexity.

Undoubtedly, there could be a coincidence between the increase in the size of the brain and the improvement in markers of social intelligence over the last 3 million years (De Miguel and Henneberg, 2001), though it is not obvious which is the cause and which the effect. However, recent work has shown that an increase in hominin brain size parallels increasing in hominin body size (Henneberg, 1998; Henneberg and Saniotis, 2009; Saniotis and Henneberg, 2011). This infers that these changes in brain volume may be a result of increasing body size rather than a reflection of increasing intelligence. The increase in body size itself may be a result of increasingly better management of resources by hominins (Olney et al., 2015) allowing growth in body size. Better resource management may be a result of improved mental abilities due to changes in neurohormonal regulation and brain physiology.

Furthermore, the concept of a larger brain leading to better cultural and technological performance did not hold true for the Holocene (last 10,000 years), when the average human brain size decreased by approximately 10% (100–150 ml, that is one standard deviation; Henneberg, 1988; Brown, 1992; Ruff et al., 1997). This is the period during which time civilizations developed, written language and formal mathematics were introduced and sciences and technologies progressed (Henneberg and Steyn, 1993).

## Human and Animal Brain: What the Research Shows

**Figure 1:**
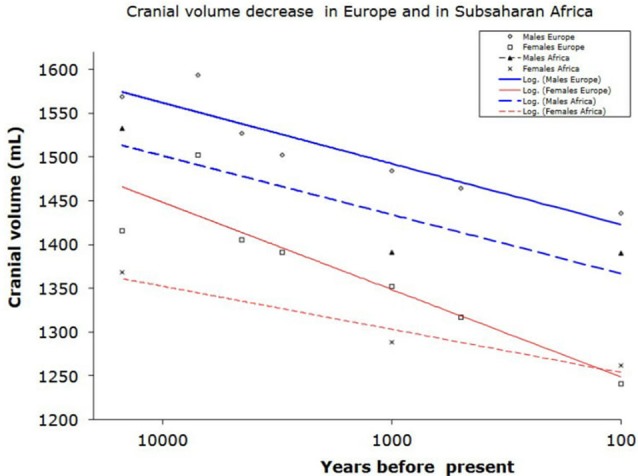
Reduction of braincase volume in the last 10,000 years in Europe (including Mediterranean) and in Subsaharan Africa. Data from Henneberg (1988) and Henneberg and Steyn (1993).

Behavioral differences between apes and humans and advances in the history of human evolution can be adduced to superior chemical regulation of brain function rather than larger brain size (see Figure 1).

There is evidence to support this postulation, with humans demonstrating 3-fold higher thyroid hormone activity and more developed dopaminergic system in the pre-frontal cortex in comparison to other primates (Previc, 1999; Gagneux et al., 2001). While Gagneux et al. (2001) postulate a possible link between lower T3 and T4 in chimpanzees with hypothyroidism and associations with cretinism, this does not explain how chimpanzees in captivity have consistently been shown to possess far better working memory of numbers than humans (Inoue and Matsuzawa, 2007). The hypothesis of superior chemical brain regulation leading to better cognition has not yet been falsified. Contrariwise, the wide use of substances that alter brain functions in humans shows a number of positive and negative influences upon cognitive abilities.

A considerable body of evidence that detracts from the significance of brain volume in relation to intelligence can be derived from the broader animal kingdom. Measuring intelligence across different species is an elusive exercise and fraught with multiple problems. One such problem is the misnomer of the uniformity of the cerebral cortex in mammalian species (Elston and Garey, 2009). This prevalent viewpoint assumes that mammals share the same cortical organization (Kolb and Tees, 2000; Jerison, 2001; Elston and Garey, 2009). However, a counter-argument notes that acceptance of the cortical uniformity hypothesis refutes heterogeneity in cortical microstructures (pyramidal neurons in the granular level of the PFC) at both inter and intra levels in mammals (Elston, 2003; Elston et al., 2006). Fuster (2001) points out the fallacy of one specific function attributed to the PFC while ignoring other complementary functions.

It has been shown that pyramidal cells are not only integral to basic cortical synaptic excitation and inter-areal and intra-areal projections but also reveal remarkable heterogeneity in the primate cerebral cortex (Elston et al., 2011). The variation in pyramidal cells in the cortices in different species demands a less essentialistic approach (Kasper et al., 1994; Gao and Zheng, 2004; Spruston, 2008). At this stage, our understanding of neuronal diversity in all of its complexity is still underdeveloped.

Most of the phylogenetic analysis of pyramidal cells have derived from the primary sensory cortices of laboratory mice (Luebke, 2017). Consequently, our understanding of pyramidal cell heterogeneity from a large range of mammalian species is lacking. For example, the dynamic processes of mouse cortical neurons (consisting of multimodal FC and V1 that are low in their dynamic range) are suitable for quick synaptic communication (Luebke, 2017). However, the neocortex of mice is less differentiated and less complex than the cortex of rhesus monkeys. The greater complexity of the latter requires more functionally marked cortical areas that are qualitatively different in their levels of filtering, excitation and input integration than mice cortical areas (Barbas, 2015; Luebke, 2017).

For the purposes of discussion, animal intelligence has been broadly defined as the ability to produce flexible responses to challenges posed by the environment and by other individuals. It is generally accepted that larger animals possess larger brains and many of these species are associated with higher intelligence (Jerison, 1973). However, mammals possess a large range of brain sizes and body sizes of approximately “eight orders of magnitude” (Dicke and Roth, 2016). Generally speaking, both mammal and avians have brains that are approximately 10 times larger than those of vertebrate fish, amphibians and reptiles of equivalent body mass (Lefebvre, 2012). Yet, bird cortices are smooth and very small while birds’ behaviors match those of many mammals and humans (e.g., language communication). Most of the complex neuronal processing in birds’ brains occurs in subcortical structures, especially in the dorsal ventricular ridge that may perform the same functions as the mammalian neocortex (Font et al., 2019). This clearly indicates that the same phenotypic characteristics of “intelligent” behaviors can be facilitated by different parts of the brain with different interconnections. The size or localization of brain structures underlying behaviors is of little consequence.

Although primate cortical cell densities are comparatively higher than those found in cetaceans and elephants, this does not explain why primate brains are superior in relation to intelligence. Neuronal densities in the human cortex are much lower than those in great apes (Haug, 1987) While there is a strong general correlation between brain size and body size, there is no such correlation among sub-samples of closely related species where the range of unexplained variation of brain/body size relationships is large (Dicke and Roth, 2016). This becomes evident when comparing relative brain size between animal species even after correcting for body size. Although parrots (*psittacids*) possess larger brain volume than corvids, both show high intelligence and have the ability to manipulate objects as tools (Dicke and Roth, 2016). Similarly, dolphins possess a bigger “corrected value” brain size than chimpanzees and gorillas but are considered by some theorists as being less intelligent than Pongidae (Manger, 2013; Güntürkün, 2014; Dicke and Roth, 2016).

**Figure 2:**
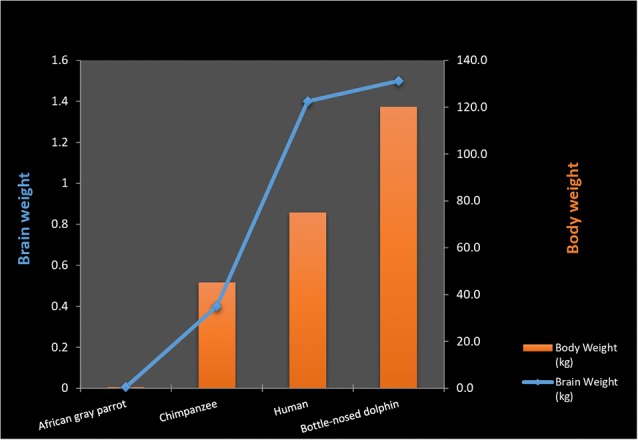
Body weight to brain ratio in various animal species.

Both the smallest and the largest mammals (shrews, mice, elephants, and whales) have large repertoires of flexible behaviors. Despite these factors, a general association between the encephalization quotient and complex behavior among mammals has been postulated (Geary, 2005). However; complications are encountered when stepping outside the mammalian class. Birds generally possess encephalization coefficients (Armstrong and Bergeron, 1985), and cortical volumes that are inferior to those of most mammals (Lefebvre, 2013; Roth, 2013). Despite this, they display enormous behavioral complexities, including the use of tools, construction of complex nests, and “intelligent” conversation in human languages. A recent study concludes that three species of Corvidae (New Caledonian crows, jackdaws, ravens) have the motor regulation equivalent to great apes despite having much smaller brains (Kabadayi et al., 2016; Figure 2).

It has also been shown that ravens (*Corvus* *corax*) recognize what conspecifics can be accessed or not—a rudimentary “Theory of Mind” (Emery and Clayton, 2001; Dally et al., 2006; Bugnyar et al., 2016). So why are some avian species so smart? It seems that the avian brain has undergone convergent evolution where it does not follow the six-layered PFC as found in mammals (Emery and Clayton, 2005). Instead, the avian homolog of the PFC could be the caudolateral nidopallium. This area shares similarities with a mammalian prefrontal cortical function such as delayed task response, working memory and reversal learning (Emery and Clayton, 2005). The architecture of the avian brain reveals how nature has been able to evolve intelligent animals with small brain volume when compared to non-human and human primates.

## Neurotransmitters and Neurotrophins in Human Brain Evolution

### Dopamine

There is increasing evidence to support the importance of neurotransmitter regulation and its impact on brain development and intelligence during hominin evolution (Raghanti et al., 2010). Various authors have described the role of DA in human brain evolution (Previc, 2002, 2009; Raghanti et al., 2010). DAergic innervation is involved in motor planning, and higher-order cognitive abilities including reasoning, language comprehension, future projection and general intelligence (Raghanti et al., 2010).

Humans and other primates receive DA through many cortical regions. The human brain not only has increased DAergic efferent density in layers III, IV, and VI PFC areas, but has more DAergic input to the PFC regions than other primates (i.e., chimpanzees, macaques; Akil et al., 1999).

The idea of neurohormonal regulation of DA acting as a factor that guided primate evolution has been investigated (Previc, 2009). The increase in DA concentrations has paralleled increases in the size of the human neocortex (Raghanti et al., 2008). Previc formulated the DA hypothesis. This hypothesis is posited on climatic changes, which occurred during the Pliocene/Pleistocene transitions (~2 Ma ago) leading to changes in hominin morphology that were positively selected for endurance hunting/foraging over a large ecological range (Previc, 2009). Thermal stress resulting from endurance hunting triggered thermoregulatory mechanisms involving DA (Roelands and Meeusen, 2010). This idea concurs with Hoffmann (2013) who suggested that DAergic dependent cognitive faculties are an exaptation of DA mediated thermo-regulatory function. *Previc’s theory* is supported by recent findings which indicated that a variation of the gene cluster in chromosome 11 (i.e., that converts fatty acid desaturase cluster or FADS) occurred ~85 ka, a time when hominins may have increased their intake of omega 3 DHA sea food, thereby influencing human brain evolution (Mathias et al., 2012). Paleoanthropological findings confirm that ancestral hominins in Pinnacle Point, South Africa (~160 ka; Marean et al., 2007), Eritrea (~125 ka; Walter et al., 2000), and the South African coastline (~100 ka; Broadhurst et al., 2002) included shellfish in their diet.

**Figure 3:**
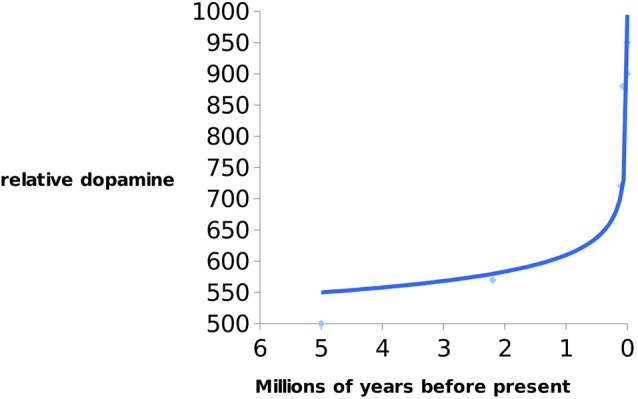
Indication of increase of dopamine (DA) levels in hominins over 5 Ma till present. Data from Previc (2009).

*Previc* argues that an increase in DHA may have consequently increased the levels of DA and thyroid hormone, thyroxine (T4), which is necessary for expanding human cognition (i.e., working memory, language fluency, and creativity). Interestingly, it is known that humans possess significantly higher concentrations of T4 than chimpanzees, as discussed earlier. This hormone is responsible for converting tyrosine to L-dopa (DA precursor). Numerous studies have confirmed that deficiencies in DA are commonly associated with neuro-cognito-behavioral impairments in humans (Previc, 2002; Brisch et al., 2014; see Figure 3).

According to Previc’s thesis DA increase in the last 9,000 years was approximately the same as during the last 8 million years. It is interesting that exponential increase in DA happened during the early Neolithic period (10 ka ago)—when there was a transition from foraging/hunting to agricultural societies/animal husbandry. It is also evident that DA in *Homo* has been steadily rising since the early Neolithic period to the present accompanying the reduction of brain size.

### Neurotrophins

Several studies have also linked neurotrophins with increasing cognitive abilities during human evolution (Raichlen and Polk, 2013). Raichlen and Polk (2013) argue that increasing physical activity levels (PAL; i.e., persistent hunting) in *H. erectus* onwards upregulated neurotrophin and growth factors prodution, such as brain derived neurotrophic factor (BDNF), vascular endothelial growth factor (VEGF), and Insulin like growth factor (IGF). This upregulation led to increasing hippocampal neurogenesis, planning abilities, spatial memory and neuroplasticity (Mattson, 2012; Noakes and Spedding, 2012; Raichlen and Polk, 2013). This transition to higher PAL, may, therefore, have accelerated human cognitive abilities. Indeed, this transition to higher PAL would have come at a time of other socio-biological developments in *Homo* such as extended adolescent period, increasing reliance on technology for food procurement and security and increasing social complexity (Saniotis and Henneberg, 2013b).

Therefore, BDNF may have been positively selected due to its neurotrophic and neurogenetic functions. Such probable positive selection may explain the correlation between exercise and BDNF production, and why exercise is important in neurogenesis in the hippocampal dentate gyrus, and enhanced cognitive performance (Saniotis and Henneberg, 2013b). In one animal model, mice were given an extensive running regime in order to ascertain the correlation between cortical growth factor production with increasing PAL. It was found that the exercised mice had increasing neurogenesis in the hippocampus—a function of VEGF (Gómez-Pinilla et al., 1998).

## Enteric Gut Microbiome and Neuro-Hormonal Regulation

### Enteric Gut Microbiome Complexity

It is becoming increasingly more likely that complex neuro-hormonal regulation is crucial in the optimization of neuronal performance. This control is provided within the central nervous system and also from elements arising outside the brain, including the gut. Micro-organisms have been co-existing in the gastrointestinal tract of metazoans in a symbiotic relationship (Cho and Blaser, 2012; Mayer et al., 2015). The human intestinal tract alone consists of approximately 100 trillion (10^14^) microbes, and the gut reservoir of all extant humans is between 10^23^ and 10^24^ microbial cells (Ley et al., 2006; Table 1). Humans are not individual entities but entangled communities of cells and microbes which maintain all body processes including human cognition (Gilbert et al., 2012; Lorimer, 2016; Nading, 2016; Rees et al., 2018).

**Table 1:** Bacterial species which produce key neuro-metabolites.

Bacteria	Neuro-transmitter/Neuro-modulator produced
Enterococcus spp.	Serotonin
Escherichia	
Corynebacterium spp.	
Streptococcus spp.	
Bacillus spp.	Dopamine
Lactobacillus spp.	Acetylcholine
Lactobacillus spp.	γ-aminobutyric acid (GABA)
Bifidobacterium spp.	
Dobacterium	
Echerichia spp.	
	Noradrenaline
Bacillus spp.	
Saccharomyces spp.	

*Data from Roshchina (2010), Barrett et al. (2012), Matur and Eraslan (2012), Lyte (2013) and Dinan et al. (2015)*.

The EGM in humans plays host to thousands of bacterial and protozoan species, thus containing 100 times more genes than their human hosts, and is a very active site for gene encoding (Ezenwa et al., 2012). For this reason, the EGM has been referred to as the meta-genome (second genome) that may be viewed as an essential organ (Zhao, 2010). Since the human enteric nervous system contains 500 million neurons (Furness, 2006), there is an ample opportunity for interactions between the gut biome and the nervous system.

### CNS and EGM Communication

The ability for the EGM to communicate with the human brain *via* the gut-brain-enteric-microbiota-axis (GBEMA) in order to maintain symbiotic homeostasis is a crucial aspect of neurohormonal regulation and plays a pivotal role in human cognition (Rhee et al., 2009; Mayer, 2011; Carabotti et al., 2015; El Aidy et al., 2015).

A number of studies indicate that the increasing use of antibiotics since the 1970s and 1980s may have led to a reduction of beneficial gut flora, thereby compromising digestive function and altering the production of neurotransmitters (DuPont and DuPont, 2011). Importantly, compromised EGM has recently been shown to contribute to many psychiatric and immunological disorders (i.e., asthma, arthritis, autism, Celiac disease, eczema, general allergies; Bested et al., 2013; Mawe and Hoffman, 2013). It should be noted that gut bacterial species also play an important role in neonatal brain development by modulating neurotrophins such as BDNF, thereby triggering synaptogenesis (Douglas-Escobar et al., 2013). Research in germ-free animals suggests that EGM colonization affects neural circuitry and behavior (Bercik et al., 2011; Cryan and Dinan, 2012; Davidson et al., 2018).

Other findings indicate that neonatal exposure to animals that have microbial pathogens may lead to reduced cognitive function and anxiety phenotypes (Diaz Heijtz et al., 2011; Carabotti et al., 2015; Kelly et al., 2015; Davidson et al., 2018). In another recent study, germ-free mice which had changes to their gastrointestinal bacterial colonies had lower expression of proteins PSD-95 and synaptophysin, which are involved in synaptogenesis (Diaz Heijtz et al., 2011). It has also been shown that a transplanted mouse strain of the fecal microbiome resulted in the recipient mouse exhibiting behaviors that were synonymous to the host mouse (Collins et al., 2013; Foster et al., 2016).

Alterations to the EGM may lead to intestinal dysbiosis resulting in faulty communicative pathways in the GBEMA, which in turn may contribute to disturbed central nervous system function (Diaz Heijtz et al., 2011; Cryan and Dinan, 2012; Mohajeri et al., 2018). It is interesting to note that partial removal of the vagus nerve (central to Gut-Brain communication), or vagotomy, annuls the probiotic effect of the EGM (Cryan and Dinan, 2012). This finding suggests that neurometabolites that are produced in the EGM are vagus-dependent (Cryan and Dinan, 2012).

Similarly, administration of probiotics in the form of *Bifidobacteria infantis* into naïve rats was associated with an attenuation of pro-inflammatory response (IFN-γ, TNF-α and IL-6 cytokines) and an increase in the neurotransmitter serotonin, which is key to mood regulation and learning, and therefore, crucial to cognitive organization (Desbonnet et al., 2008). A possible reason for this could be because *Bifidobacteria infantis* is one of the most prolific bacterial genera within the first days to weeks of the neonate (Boesten et al., 2011; Jost et al., 2012; Underwood et al., 2015).

Indigenous strains of microbiota have also been found to modulate serotonin in the hippocampus, as well as, regulating serotonin host biosynthesis (Clarke et al., 2013; Yano et al., 2015).

### EGM Modulation by Vagus Nerve and Cognition

Some researchers contend that the “microbiota-gut-vagus-brain axis” may modulate human behavior (Montiel-Castro et al., 2013; Alcock et al., 2014), while other studies note that some “subliminal interoceptive” gut inputs from microbiota may influence human affective states, memory formation, and decision-making processes (Craig, 2002; Berntson et al., 2003). Information from the gut to the vagus nerve is transmitted to the brain where it is processed in the nucleus solitarius which assists in homeostatic regulation, with other projections to the amygdala and PFC (Mohajeri et al., 2018). The amygdala is an area for microbial induced gene activity (Morgan et al., 1993; Stilling et al., 2015). Microbiotic modulation of the vagus nerve was demonstrated in mice in which the introduction of a specific gut bacterial species led to the triggering of vagus nerve dependent neuronal regions in the brain, leading to anxiety type behavior (Goehler et al., 2005; Foster et al., 2016). These findings point to neuro-hormonal regulation outside the cerebrum as a factor in affecting human cognitive abilities. It has been suggested that a reason for EGMs influence of the CNS is due to gut bacterial ability to produce neurochemicals that are structurally similar to the host’s nervous system (Lyte, 2013). This bio-mimicry faculty of gut bacterial species implies that there exists a constant bidirectional communication between the EGM and host.

### GI Tract Inflammation and Cognition

Individuals with intestinal disorders are often affected by cognitive and mood disorders. For example, MRI brain imaging of children (10–14 years old) with Crohn’s disease showed thinning of the posterior and middle frontal gyri, poorer cognitive and verbal memory and impaired performance (Berrill et al., 2013; Gareau, 2016; Mrakotsky et al., 2016). There has also been found a correlation between colonic inflammation and deficits in the CA1 region of the hippocampus (Novotný et al., 2019). Research studies have drawn correlations between the EGM and neurological and psychiatric disorders such as schizophrenia, multiple sclerosis, Parkinsons’ disease and Alzheimer’s disease (Fröhlich et al., 2016; Parashar and Udayabanu, 2016; Martin et al., 2018; Novotný et al., 2019). Inflammation of the GI tract can compromise the gut barrier function (leaky gut syndrome) and enable bacteria can pass into the systemic circulation. Such translocation of bacteria has been linked to pathophysiologies and altered cognition (Giannelli et al., 2014; Slyepchenko et al., 2017; Hegde et al., 2018; Sarkar et al., 2018).

### Cognition and EGM: Prenatal and Postnatal Brain Development

Studies conducted on the microbiome in the early life stage have identified that gut bacteria takes approximately 2–3 years before it resembles the adult microbiome (Yatsunenko et al., 2012; Goyal et al., 2015; Carlson et al., 2018). Rapid neurophysiological development of neonates is accompanied by equally prompt changes of the microbiome. This concomitant growth could be important in order for the microbiome to regulate metabolic processes of the neonate brain (Al-Asmakh et al., 2012; Koren et al., 2012). The nascent influence of the microbiome has profound effects through human ontogeny. Several studies argue that gut bacteria can infiltrate the meconium, amniotic fluid and placenta (Jiménez et al., 2008; Gosalbes et al., 2013; Hansen et al., 2015; D’Argenio, 2018). The placenta may also produce serotonin (5-HT) to reach the fetus. Serotonin is important for development of the fetal forebrain (Al-Asmakh et al., 2012; Goyal et al., 2015). During fetal development, the placenta regulates fetal stress response by hormonal interacting with the hypothalamus-pituitary-adrenal (HPA) axis (Al-Asmakh et al., 2012). Rat models reveal that HPA activity in the fetus may lead to a decline in cognitive performance and elevated behavioral and anxiety response (Alonso et al., 1991; Vallée et al., 1997). Although the current understanding of prenatal development is increasing, researchers have yet to determine the microbiotic mechanisms informing the fetal brain. In the future microbial communities could be used to support fetal cognitive development (Carlson et al., 2018).

### Neuroendocrine System and Cognition

At least 12 types of neuroendocrine and enteroendocrine cells (NECs) are located in the epithelial cells of the GI tract and are involved in more than 20 molecular signals (Furness et al., 2013; Martin et al., 2018). Changes in the EGM can lead to subsequent changes to neurotransmitters and neuropeptides (Novotný et al., 2019). Various authors note that molecular neuropeptides and neurotransmitters (acetylcholine, GABA, DA, melatonin histamine) are activated by EECs (Barrett et al., 2012). The EGM modulates serotonergic pathways between the CNS and ENS which assist in learning and cognition (Mahoney et al., 2005; Forsythe et al., 2016). The importance of neuroendocrine regulated 5-HT has been the focus of several studies. One study in which mice were colonized by human fecal microbiota resulted in an increase in 5-HT tissue concentration colonic tryptophan 5-hydroxylase 1 (TPH1; Reigstad et al., 2015). Furthermore, the bacterial species *Clostridia* promotes the biosynthesis of 5-HT by enterochromaffin cells (ECs; Forsythe et al., 2016). It has been speculated that EC derived 5-HT may influence EGM composition with subsequent changes in brain regulation and behavior (Forsythe et al., 2016).

## Conclusion

The hypothesis presented here argues that brain size does not exert an influence on an individual’s cognitive abilities. Even the most ardent supporters of the correlation between brain size and intelligence confirm that brain size contributes to no more than approximately 10% of the total variance in intelligence. Moreover, this level of correlation may be a result of the correlation of both variables to some third factor (e.g., body size) rather than a direct relationship. Any small-headed, highly intelligent person (and there are millions of them) falsifies the brain size-intelligence relation hypothesis. It is well known that correlation does not prove causation. The findings of many investigations have indicated the importance of neurotransmitters in the regulation of cognitive function. DA, 5HT, and neurotrophins have been identified as the important neurotransmitters that regulate this neurological performance. Evolutionary evidence supports the involvement of DA, 5HT and neurotrophins in the development of the human brain. Furthermore, the current use of neurotransmitter agonists or antagonists in the treatment of various conditions associated with neurotransmitter imbalances implicates neuro-hormonal regulation as a key factor influencing neurological performance of the brain, particularly intelligence. Drug addictions show the obvious influence of chemical regulation on brain function. In addition, neurotransmitters that are synthesized by the enteric microbiome appear to affect changes in cognitive aptitude, namely intelligence. Moreover, this article has highlighted the influential role of the EGM in informing human cognition.

Technological advancements will likely facilitate a more intricate approach to detailing the components that dictate intelligence. The evidence presented in this article suggests that the paradigm of what influences intelligence needs to be reassessed once more. It appears that the archaic practice of considering that mental fortitude is dictated by brain volume is beginning to recede. A more insightful approach, taking into account the concentration and regulation of neurotransmitters, as well as, the synaptic architecture of the brain is necessary if our understanding of intelligence is to advance. The potential for other, as yet unknown factors, playing a role also needs to be considered.

## Data Availability Statement

Publicly available datasets were analyzed in this study. This data can be found here: Henneberg (1988), Previc (2009), Roshchina (2010), Barrett et al. (2012), Matur and Eraslan (2012), Lyte (2013) and Dinan et al. (2015).

## Author Contributions

AS: substantial contributions to the conception or design of the work, drafting the work or revising it critically for important intellectual content, provide approval for publication of the content and agreed to the accuracy or integrity of the article. JG: substantial contributions to the conception or design of the work and agreed to the accuracy or integrity of the article. JK and MH: contributions to the conception or design of the work and agreed to the accuracy or integrity of the article.

## Conflict of Interest

The authors declare that the research was conducted in the absence of any commercial or financial relationships that could be construed as a potential conflict of interest.
